# Supra-pharmacological concentration of capsaicin stimulates brown adipogenesis through induction of endoplasmic reticulum stress

**DOI:** 10.1038/s41598-018-19223-2

**Published:** 2018-01-16

**Authors:** Ryosuke Kida, Taiki Noguchi, Masaru Murakami, Osamu Hashimoto, Teruo Kawada, Tohru Matsui, Masayuki Funaba

**Affiliations:** 1 Division of Applied Biosciences, Graduate School of Agriculture, Kyoto University, Kyoto, 606-8502 Japan; 2 Laboratory of Molecular Biology, Azabu University School of Veterinary Medicine, Sagamihara, 252-5201 Japan; 3 Laboratory of Experimental Animal Science, Kitasato University School of Veterinary Medicine, Towada, 034-8628 Japan; 4 Division of Food Science and Biotechnology, Kyoto University Graduate School of Agriculture, Uji, 611-0011 Japan

## Abstract

We previously showed that brown (pre)adipocytes express Trpv1, a capsaicin receptor, and that capsaicin stimulates differentiation of brown preadipocytes in the late stages of brown adipogenesis. The present study revealed that treatment with 100 μM capsaicin stimulates brown adipogenesis by inducing endoplasmic reticulum (ER) stress. Treatment with capsaicin (100 μM) during brown adipogenesis enhanced lipid accumulation and the expression of Ucp1, a gene selectively expressed in brown adipocytes. Capsaicin treatment also caused an increase in the cytosolic calcium concentration even when extracellular calcium was removed. I-RTX, a Trpv1 inhibitor, did not modulate the increase in cytosolic calcium concentration, lipid accumulation or Ucp1 expression. Previous studies revealed that the release of calcium from the ER induces ER stress, leading to the conversion of X-box binding protein 1 (Xbp1) pre-mRNA to spliced Xbp1 (sXbp1) as well as the up-regulation of Chop expression. Capsaicin treatment increased the expression of sXbp1 and Chop in brown preadipocytes and did not enhance lipid accumulation or Ucp1 expression in Xbp1 knockdown cells. The present results describe a novel mechanism of brown adipogenesis regulation via ER stress that is induced by a supra-pharmacological concentration of capsaicin.

## Introduction

Obesity, defined as an increase in body fat mass, is one of the leading causes of chronic illness and premature death; obesity triggers various diseases such as type 2 diabetes, hypertension and cardiovascular disease^1,2^. Obesity is the result of an imbalance between energy intake and energy expenditure. Therefore, efficient energy expenditure is critical for the prevention and therapy of obesity.

Brown adipocytes are one of two types of fat cells involved in energy expenditure. Upon activation of the sympathetic nervous system, brown adipocytes induce non-shivering thermogenesis^3^. To elicit thermogenic activity, brown adipocytes express β-adrenergic receptor as well as a series of genes related to mitochondrial biogenesis and the enhancement of cellular respiration. Furthermore, a gene product that is uncoupled from ATP synthesis, uncoupling protein 1 (Ucp1), is expressed in brown adipocytes^3,4^. Since the characterization of functional brown adipocytes in adult humans^5–8^, many efforts have been directed towards understanding the factors affecting brown adipocyte differentiation and activation^9,10^. However, at present, the molecular mechanisms involved in brown adipogenesis as well as dietary factors controlling brown adipogenesis and brown adipocyte function are not fully understood^9,10^.

Capsaicin is the principal pungent component of red chili peppers^11^. The ingestion of capsaicin increases energy expenditure and brown adipocyte-mediated thermogenesis^12–15^. The capsaicin-induced stimulation of brown adipocytes is thought to be mediated through the activation of sympathetic nerve activity^15,16^; the stimulation of sympathetic nerve activity by capsaicin or capsinoids (capsaicin-related molecules) has been previously established^17,18^. However, we recently demonstrated the direct action of capsaicin in brown (pre)adipocytes. We showed the expression of transient receptor potential vanilloid 1 (Trpv1), a capsaicin receptor^19^, in brown adipose tissues and demonstrated that capsaicin slightly, but directly, stimulated differentiation of brown preadipocytes in the late stage of brown adipogenesis^20^. These results prompted us to further explore the role of capsaicin in the regulation of brown adipogenesis. During these studies, we unexpectedly found that a capsaicin concentration of 100 μM strongly stimulated brown adipogenesis. Treatment of cells with 100 μM capsaicin increased the cytosolic concentration of calcium in a Trpv1-independent manner. Capsaicin treatment also induced endoplasmic reticulum (ER) stress. We also found that X-box binding protein 1 (Xbp1), a gene activated in response to ER stress^21,22^, positively regulated brown adipogenesis. The present study reveals a role for ER stress in stimulating brown preadipocyte differentiation, which shows promise as a potential target of pharmacotherapies for obesity.

## Results

### Supra-pharmacological concentration of capsaicin stimulates brown adipogenesis

We previously revealed that treatment with 0.1 μM capsaicin slightly, but directly, stimulated differentiation of brown preadipocytes during the late stages of brown adipogenesis. However, this concentration did not affect brown preadipocytes during the early stages of brown adipogenesis^20^. In addition, treatments of up to 10 μM capsaicin during brown adipogenesis did not affect differentiation^20^. We evaluated whether much higher doses of capsaicin affect brown adipogenesis. At first, we examined whether the high concentrations of capsaicin induce cell death. Cell viability assay revealed that treatment with capsaicin up to 100 μM for 2 days did not decrease the number of viable cells (Supplementary Fig. 1A). On the contrary, treatment with actinomycin D (Act D) for 1 day decreased cell viability; Act D is a potent inducer of apoptosis in a variety of cells^23–25^. We also evaluated expression of full-length of poly(ADP-ribose) polymerase (Parp)-1 (Supplementary Fig. 1B), because apoptosis induces cleavage of Parp-1^26^. Expression of full-length Parp-1 was not affected by treatment with capsaicin at 100 μM, but treatment with 300 μM capsaicin decreased expression of full-length Parp-1. Furthermore, detachment of cells from culture plate, an apparent sign of cell toxicity, was not observed in cells treated with capsaicin concentrations of up to 100 μM; however, 500 μM capsaicin induced cell death within 24 hours (data not shown). Notably, when growing cells were treated with capsaicin at 100 μM, cells were detached from cell plate within 24 hours (data not shown). All these results suggest that treatment with capsaicin no more than 100 μM does not induce cell death in HB2 cells during differentiation stimulation.

**Figure 1:**
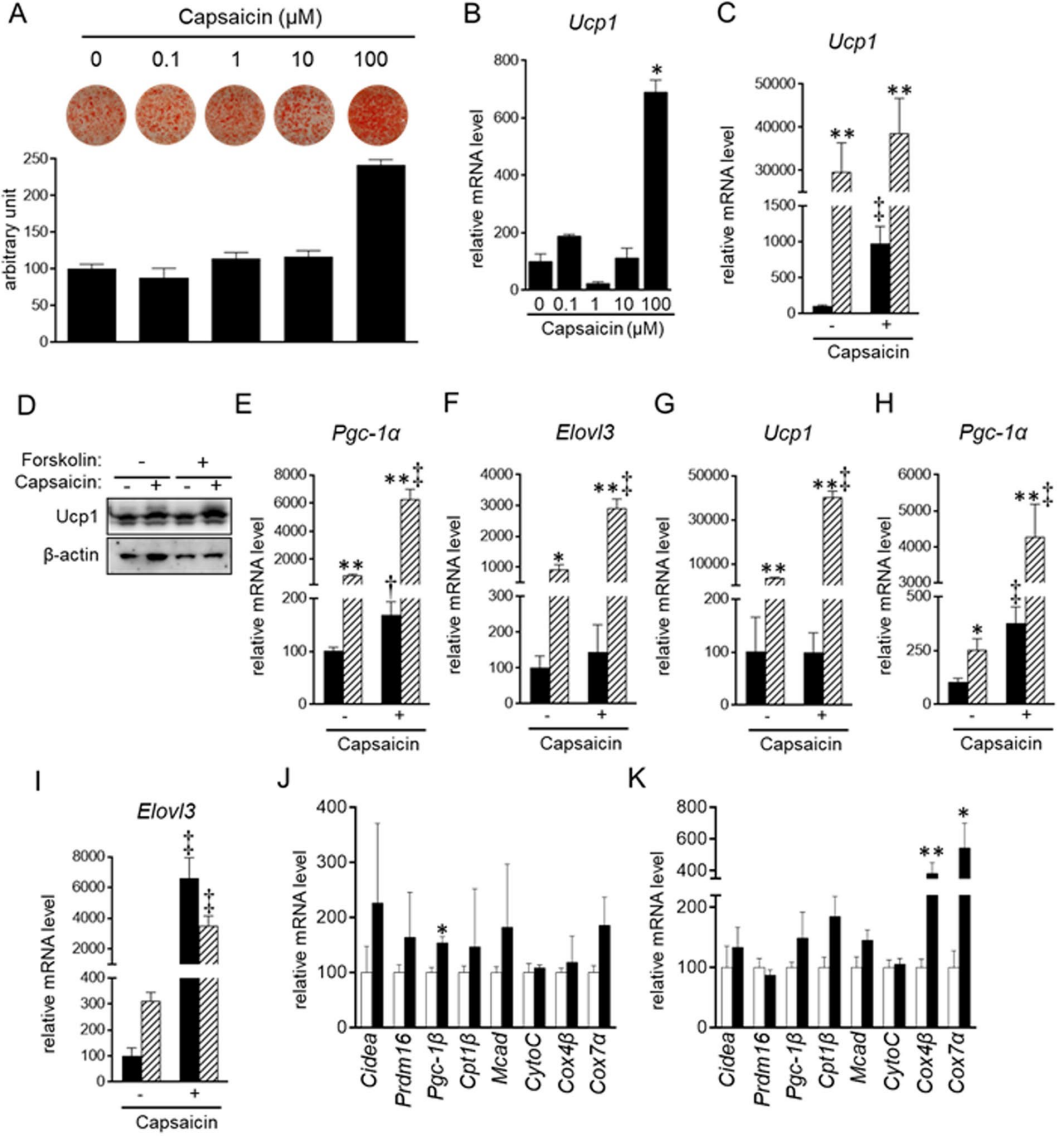
Stimulation of brown adipogenesis by supra-pharmacological capsaicin. (**A**–**F**,**J**) HB2 brown preadipocytes or (**G**–**I**,**K**) SV cells from mouse brown adipose tissues were cultured with the indicated concentrations (A and B) or 100 μM (**C**–**K**) of capsaicin during brown adipogenesis. Cells on day 8 (**C**–**F**) or day 10 (G-I) were also treated with or without forskolin (10 μM) for 4 h. (**A**) Oil Red O staining of cells on day 8 was performed and the dye intensity was quantified (n = 2). Expression levels of Ucp1 (**B**,**C**,**G**), Pgc-1α (**E** and **H**) or Elolv3 (**F** and **I**) were examined by RT-qPCR analysis. (**C**,**E**–**I**) Black bar: vehicle; Hatched bar: forskolin. (**J** and **K**) Expression levels of genes related to brown adipogenesis and function of brown adipocytes were examined in HB2 brown adipocytes on day 8 (**J**) and primary mouse brown adipocytes on day 10 (**K**) in the absence of forskolin by RT-qPCR. Open bar: vehicle; Black bar: capsaicin. The data are presented as the mean ± SE (n = 4). *P < 0.05 and **P < 0.01 *vs*. cells treated with vehicle and corresponding reagent (vehicle or capsaicin). ^†^*P* < 0.05 and ^‡^*P* < 0.01 *vs*. cells treated with vehicle and corresponding reagent (vehicle or forskolin). (**D**) Ucp1 as well as β-actin as the loading control was examined by Western blot analysis. Representative results are shown. The cropped images of Western blot analysis are shown because of space limitations; images of the full-length blot are Supplementary Fig. S6.

HB2 brown preadipocytes were differentiated in the presence of various concentrations of capsaicin. Oil Red O staining was markedly more intense in cells treated with 100 μM capsaicin than in control cells (Fig. 1A, Supplementary Fig. 2A). The color intensity correlates with the amount of dye extracted into 2-propanol, which reflects the amount of lipid content in the cell (Supplementary Fig. 2B)^27^. Thus, the intense Oil Red O staining indicated increased lipid accumulation in cells treated with 100 μM capsaicin. We also evaluated effects of 30–200 μM of capsaicin on lipid accumulation in HB2 brown adipocytes; treatment with capsaicin at < 100 μM did not significantly increase lipid accumulation (Supplementary Fig. 3). In addition, the increased lipid accumulation was also detected in cells treated with 100–200 μM capsaicin.

Expression levels of Ucp1 on day 8 was significantly higher in cells treated with 100 μM capsaicin during brown adipogenesis than in those treated without capsaicin (control cells) (Fig. 1B). Expression of Ucp1, as well as peroxisome proliferator-activated receptor-γ coactivator (Pgc)-1α, was increased in brown adipocytes in response to an increase in cytosolic cAMP concentration^28^. Elongation of very long chain fatty acids protein (Elovl)3 expression was also increased in response to treatment with epinephrine that finally increases cytosolic cAMP concentration^29^. Treatment with forskolin, an activator of adenylate cyclase that produces cAMP^30^, increased the expression of Ucp1, Pgc-1α and Elovl3 in HB2 cells; moreover, capsaicin enhanced this responsiveness to forskolin (Fig. 1C–F). Similar observations were also detected in primary brown adipocytes differentiated from stromal vascular (SV) cells of mouse brown fat; treatment with 100 μM capsaicin potentiated the forskolin-induced expression of Ucp1, Pgc-1α and Elovl3 (Fig. 1G–I). In addition, expression of Pgc-1α and Evolv3 was increased by the capsaicin in the absence of forskolin. We also examined expression of genes related to brown adipogenesis (PR domain containing 16 (Prdm16)), lipolysis and β-oxidation (cell death-inducing DNA fragmentation factor, α subunit-like effector A (Cidea), carnitine palmitoyltransferase (Cpt1)β and medium-chain acyl-CoA dehydrogenase (Mcad)), and mitochondrial biogenesis and function (Pgc-1β, cytochrome c (CytoC), CytoC oxidase (Cox)4β and Cox7α) in HB2 adipocytes (Fig. 1J) and mouse primary brown adipocytes (Fig. 1K). In general, the expression levels were numerically increased by treatment with 100 μM capsaicin; the increases in Pgc-1β in HB2 brown adipocytes and Cox4β and Cox7α in primary brown adipocytes was significant (*P* < 0.05). All these results indicated that 100 μM capsaicin stimulated brown adipogenesis. Given that the peak plasma concentration in humans ingesting capsicum containing 26.6 mg of capsaicin was 2.47 ng/ml (8.1 nM)^31^, 100 μM of capsaicin is considered super-physiological, and the effect of capsaicin is even supra-pharmacological.

**Figure 2:**
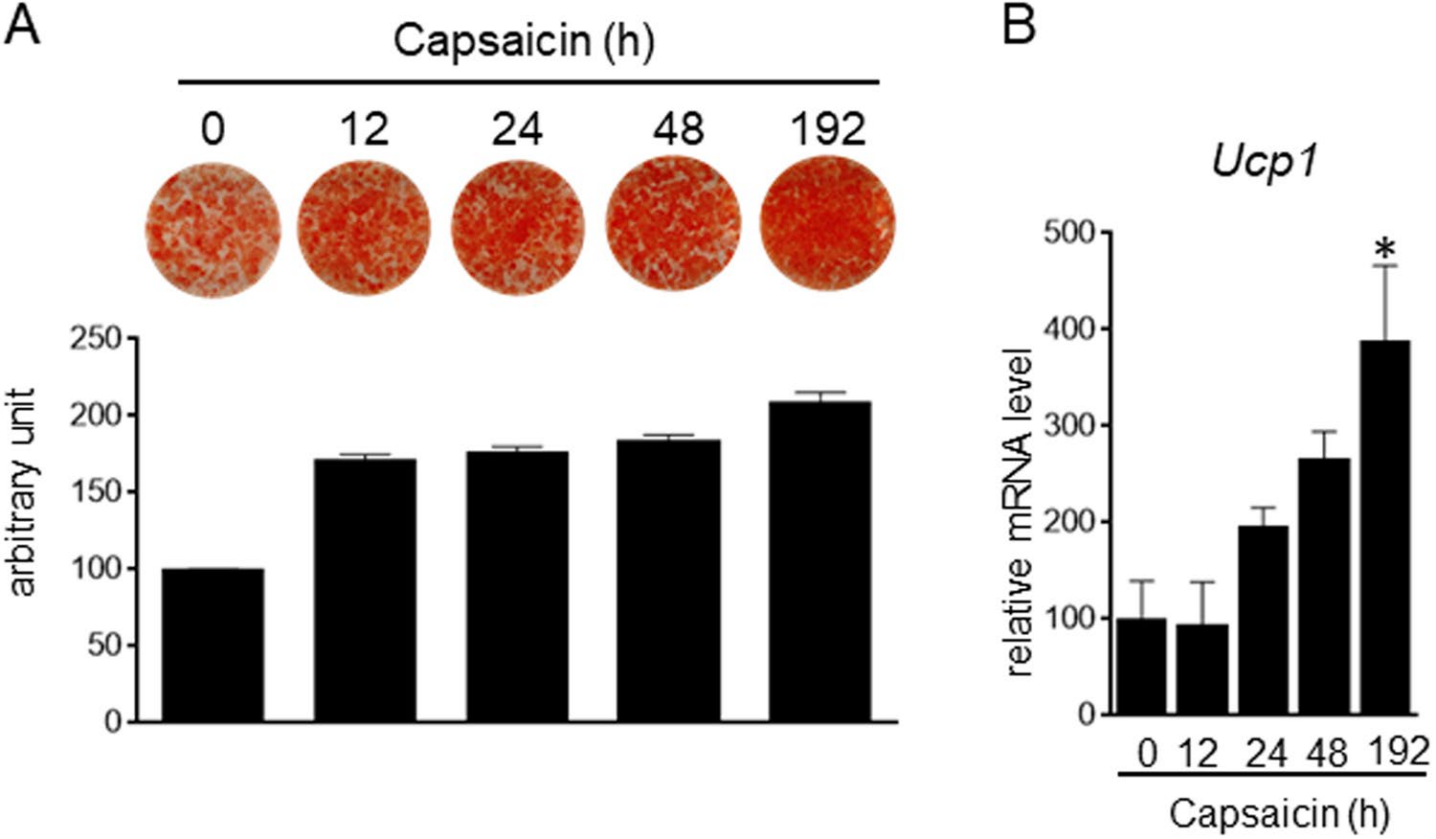
Duration required for supra-pharmacological capsaicin-induced brown adipogenesis. HB2 brown preadipocytes were treated with capsaicin (100 μM) for the indicated time. (**A**) Oil Red O staining of cells on day 8 was performed, and the dye intensity was quantified (n = 2). (**B**) The expression level of Ucp1 on day 8 was examined by RT-qPCR. The data are presented as the mean ± SE (n = 4). *P < 0.05 *vs*. control cells.

We also examined the effect of capsaicin treatment duration. Cells were treated with capsaicin at various times, and the levels of lipid accumulation and Ucp1 expression were examined on day 8 (Fig. 2). Treatment with the supra-pharmacological concentration of capsaicin for the initial 12 hours after stimulation of differentiation was sufficient to increase lipid accumulation (Fig. 2A). In addition, lipid accumulation tended to increase in cells treated with capsaicin for 8 days. In contrast, capsaicin treatment for the initial 12 hours after stimulation of differentiation was not sufficient to increase Ucp1 expression on day 8; however, Ucp1 expression was significantly increased by treatment with capsaicin for 8 days (Fig. 2B). These results suggested that the supra-pharmacological concentration of capsaicin employs multiple mechanisms to regulate brown adipogenesis.

### Supra-pharmacological concentration of capsaicin enhances efficacy of differentiation of brown preadipocytes

**Figure 3:**
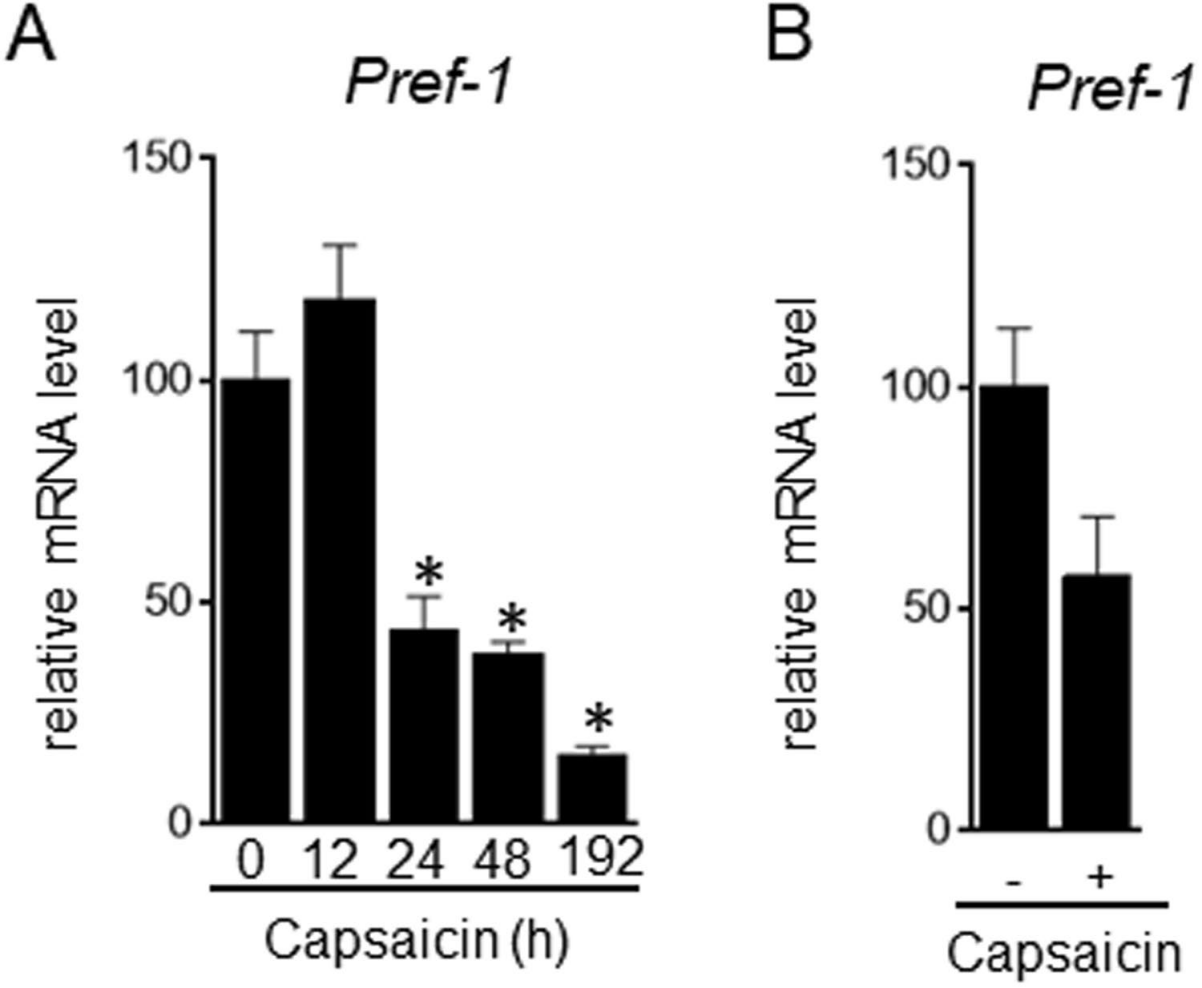
Down-regulation of Pref-1 expression in brown adipocytes treated with supra-pharmacological capsaicin. (**A**) HB2 brown preadipocytes were treated with capsaicin (100 μM) for the indicated time. (**B**) Stromal vascular cells from mouse brown adipose tissues were cultured with 100 μM of capsaicin during brown adipogenesis. The expression level of Pref-1 on day 8 (**A**) or day 10 (**B**) was examined by RT-qPCR analysis. The data are presented as the mean ± SE (n = 4). *P < 0.05 *vs*. control cells.

There are two possible explanations for the finding that supra-pharmacological capsaicin induced intense Oil Red O staining (Fig. 1A, Supplementary Figs 2 and 3): 1) efficient differentiation of the remaining brown preadipocytes during brown adipogenesis, and 2) more lipid accumulation in the capsaicin-treated brown adipocytes. Preadipocyte factor (Pref)-1, a gene highly expressed in preadipocytes, is a gatekeeper gene that acts by maintaining the preadipocyte state and preventing adipocyte differentiation^32^. Pref-1 expression on day 8 was decreased in brown adipocytes treated with supra-pharmacological capsaicin for the initial 24 hours after stimulation of differentiation, which suggested a decrease in the number of brown preadipocytes (Fig. 3A). Pref-1 expression was also decreased by supra-pharmacological capsaicin in primary brown adipocytes (Fig. 3B). We also detected cells without lipid droplets between adipocytes in the control wells (data not shown). Thus, we conclude that the supra-pharmacological concentration of capsaicin efficiently stimulates differentiation of brown preadipocytes but does not enhance lipid accumulation and Ucp1 expression in brown adipocytes.

### Supra-pharmacological concentration of capsaicin induces cytosolic calcium influx from intracellular calcium stores in a Trpv1-independent manner

**Figure 4:**
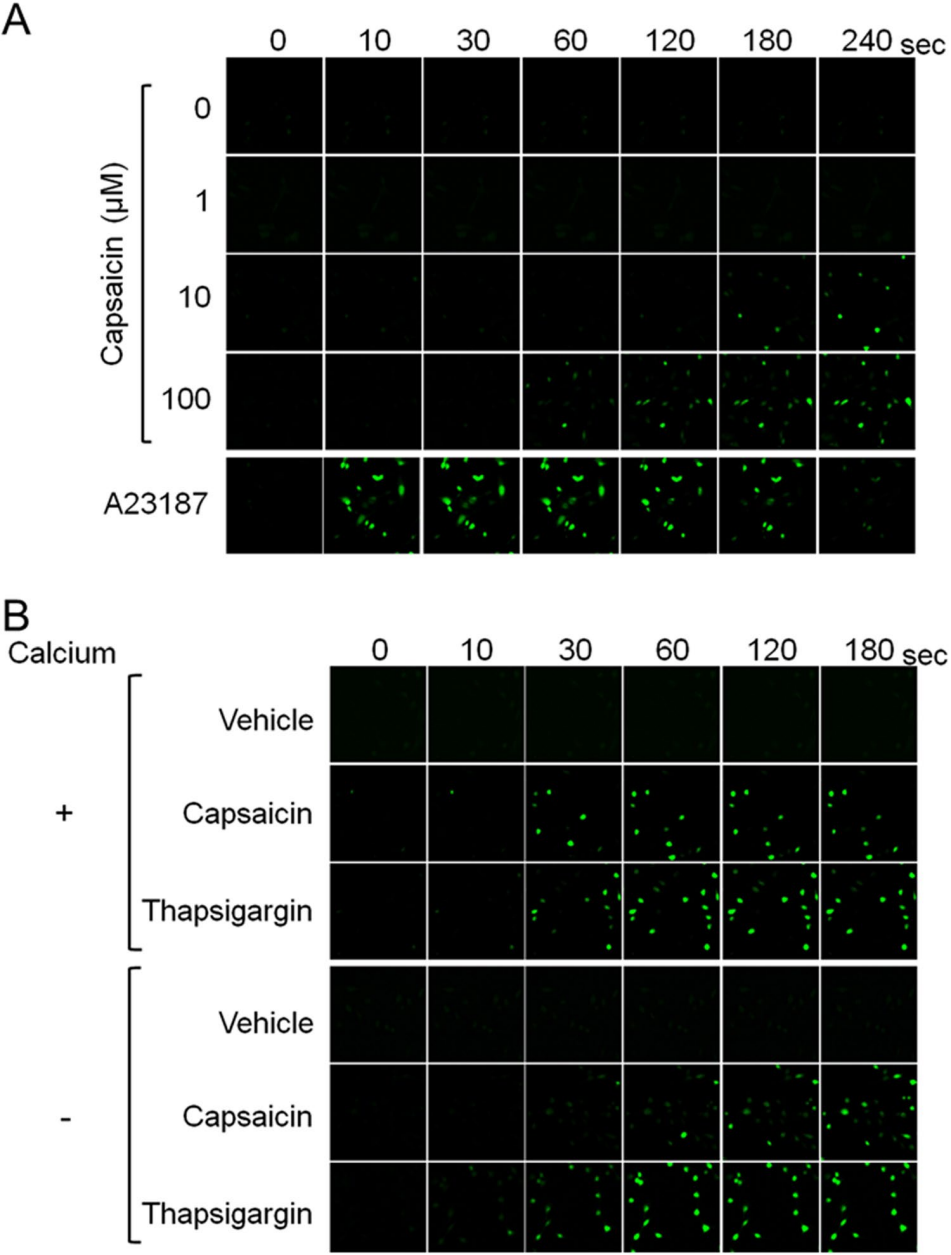
Increased cytosolic calcium level in response to supra-pharmacological capsaicin, irrespective of extracellular calcium. (**A**) HB2 brown preadipocytes loaded with Fluo-8 AM were treated with the indicated concentrations of capsaicin or A23187 (1 μM), and cytosolic calcium level was evaluated. (**B**) Effect of the depletion of extracellular calcium was evaluated. Cells were treated with vehicle, capsaicin (100 μM) or thapsigargin (100 nM).

Capsaicin increases cytosolic calcium via Trpv1^19,33,34^. We previously showed that treatment with 10 μM capsaicin increased cytosolic calcium levels in HB2 brown preadipocytes^20^. The cytosolic calcium concentration was increased within 60 sec after the treatment with 100 μM capsaicin. Compared to cells treated with 10 μM capsaicin, the increase in cytosolic calcium concentration was faster and was detected in more cells per field of view. The induced calcium influx in response to capsaicin lasted for at least 240 sec. The kinetics of calcium influx resulting from the treatment with supra-pharmacological capsaicin was different from that induced by A23187, a calcium ionophore^35^; A23187 stimulated calcium influx into cytoplasm within 10 sec after treatment but gradually attenuated after 120 sec (Fig. 4A).

Two sources of calcium potentially contribute to the rise in cytosolic calcium concentration: uptake of extracellular calcium and release of calcium stored in intracellular organelles such as the ER^36^. To evaluate the contribution of extracellular calcium, cells were treated with capsaicin in the presence or absence of calcium-supplemented medium (Fig. 4B). The increase in cytosolic calcium concentration was detected in brown preadipocytes even when extracellular calcium was absent. The increase in calcium concentration was slightly faster in the presence of extracellular calcium than in the absence of extracellular calcium, which suggested that extracellular calcium also contributed to the increase in cytosolic calcium levels induced by capsaicin. Thapsigargin inhibits sarcoplasmic/ER calcium-ATPase, resulting in an increase in cytosolic calcium concentration^37^. Thapsigargin also increased the concentration of cytosolic calcium, and extracellular calcium did not affect the kinetics of the induced cytosolic calcium concentration. These results suggested that, although the supra-pharmacological concentration of capsaicin could stimulate the uptake of extracellular calcium, supra-pharmacological capsaicin increased cytosolic calcium levels mainly through the release of intracellular calcium stores.

**Figure 5:**
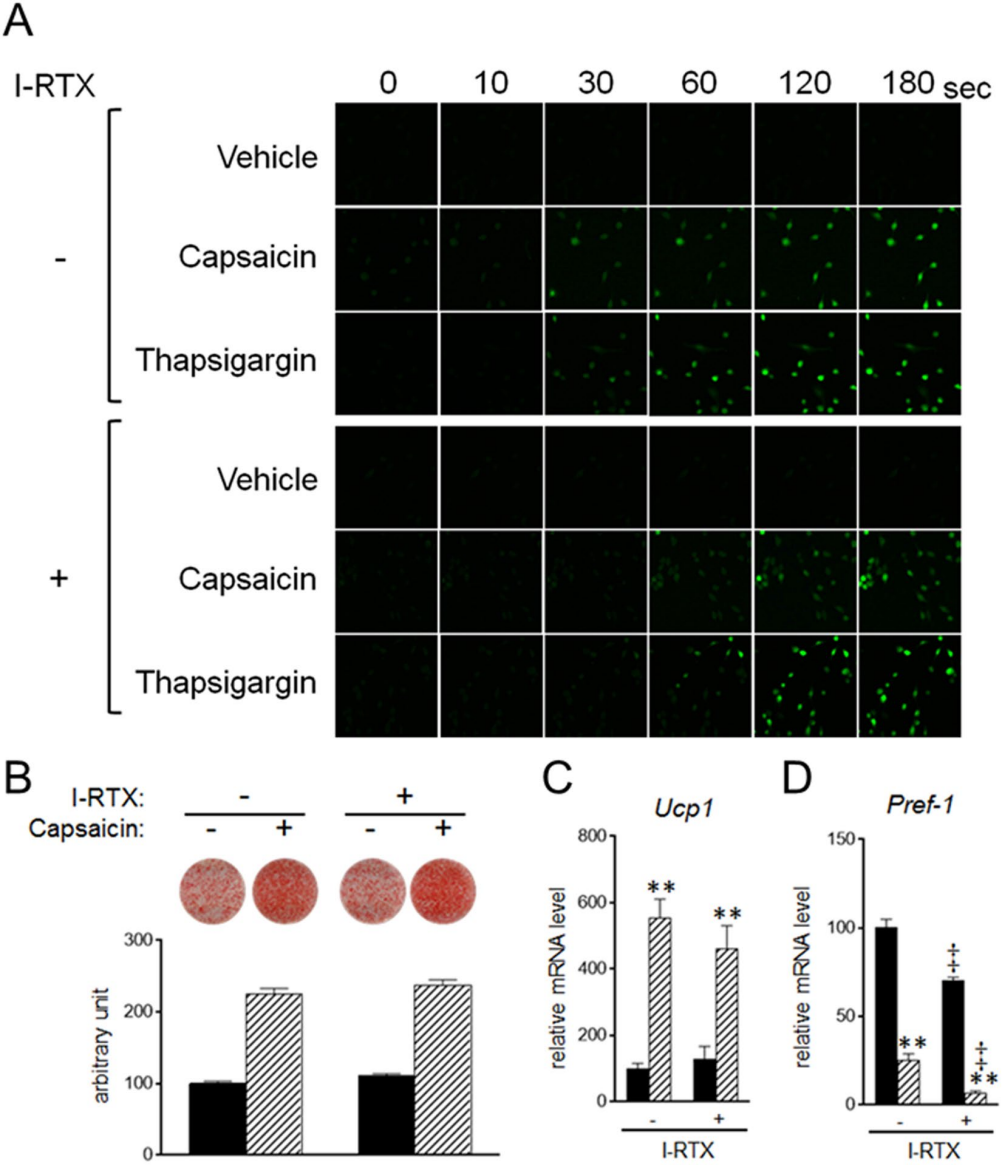
Stimulation of brown adipogenesis by supra-pharmacological capsaicin in a Trpv1-independent manner. (**A**) HB2 brown preadipocytes loaded with Fluo-8 AM were treated with capsaicin (100 μM) or A23187 (1 μM) in the absence of extracellular calcium and in the presence or absence of I-RTX (1 μM), and cytosolic calcium level was evaluated. (**B**–**D**) HB2 brown preadipocytes were cultured with 100 μM of capsaicin in the presence or absence of I-RTX (1 μM) during brown adipogenesis. (**B**) Oil Red O staining of cells on day 8 was performed and the dye intensity was quantified (n = 2). (**C** and **D**) Expression levels of Ucp1 (**C**) or Pref-1 (**D**) on day 8 were examined by RT-qPCR analysis. Black bar: vehicle; Hatched bar: capsaicin. The data are presented as the mean ± SE (n = 4). **P < 0.01 *vs*. cells treated with vehicle and corresponding reagent (vehicle or I-RTX). ^‡^*P* < 0.01 *vs*. cells treated with vehicle and corresponding reagent (vehicle or capsaicin).

5′-Iodoresiniferatoxin (I-RTX) is an antagonist of Trpv1^38^. Trpv1 is expressed on the plasma membrane as well as intracellular membranes such as the ER and mitochondria^39^. To evaluate whether the supra-pharmacological capsaicin-induced increase in cytosolic calcium concentration is mediated by Trpv1, calcium imaging was performed in the presence of I-RTX and in the absence of extracellular calcium (Fig. 5A). The capsaicin-induced increase in cytosolic calcium concentration was slightly delayed by I-RTX, but fluorescence intensity was not affected by I-RTX. I-RTX also delayed the increase in cytosolic calcium concentration by thapsigargin. It is possible that the delayed increase in cytosolic calcium concentration by I-RTX is not due to inhibition of Trpv1, and that supra-pharmacological capsaicin increased the cytosolic calcium concentration in a Trpv1-independent manner. Consistent with the inability of I-RTX to inhibit an increase in intracellular calcium, I-RTX did not affect the capsaicin-induced lipid accumulation and expression of Ucp1 (Fig. 5B,C). In addition, capsaicin decreased expression of Pref-1 even in I-RTX-treated cells; I-RTX significantly decreased Pref-1 expression, irrespective of capsaicin treatment (Fig. 5D).

### Supra-pharmacological concentration of capsaicin induces ER stress in brown preadipocytes

The calcium imaging shown above indicated that supra-pharmacological capsaicin changes the subcellular distribution of calcium. Outflow of calcium from the ER induces ER stress^36,40,41^. Thus, we hypothesized that supra-pharmacological capsaicin induces ER stress in brown preadipocytes.

**Figure 6:**
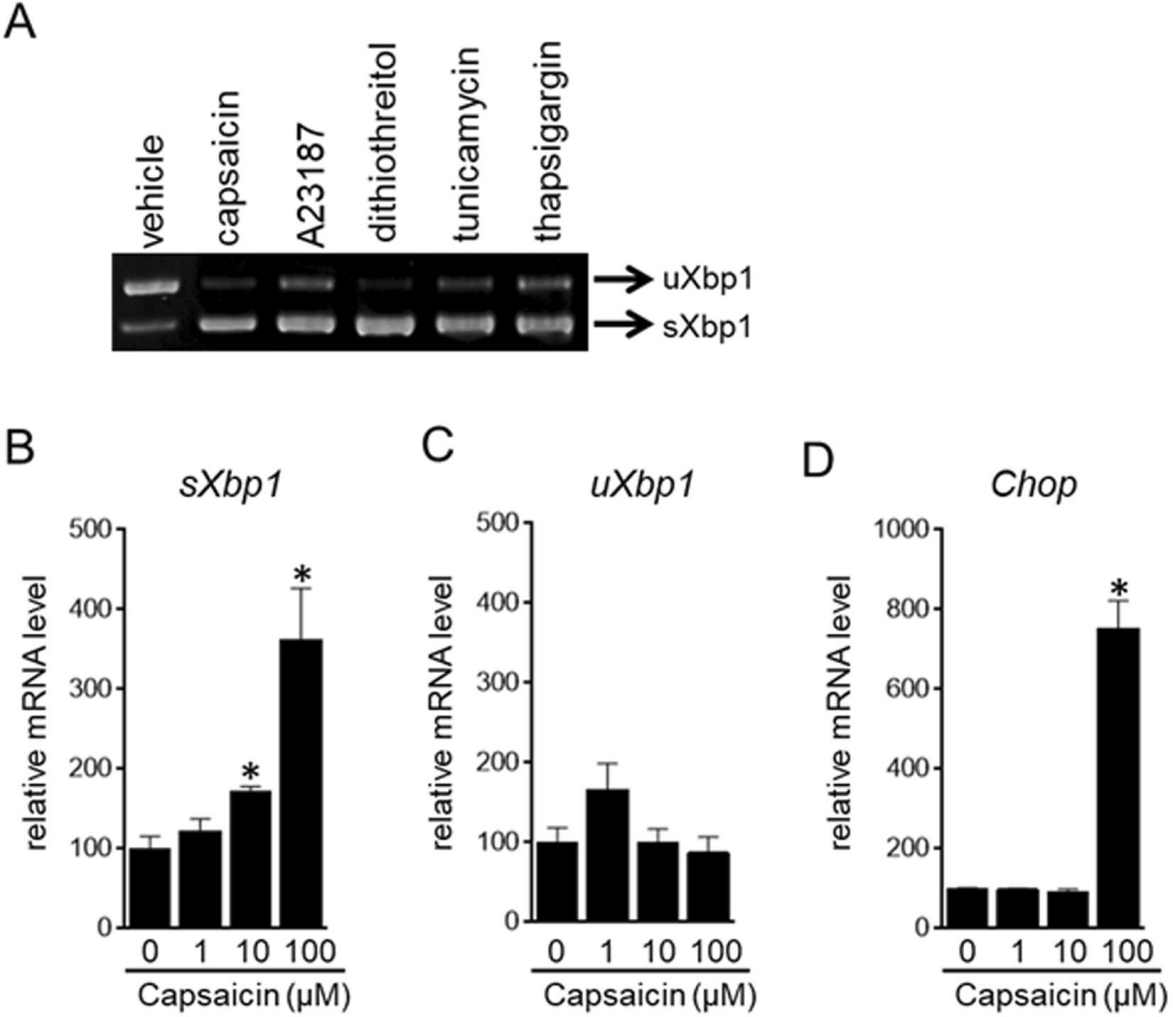
Induction of ER stress by supra-pharmacological capsaicin. (**A**) HB2 brown preadipocytes were treated with the indicated reagent, i.e., capsaicin (100 μM), A23187 (500 nM), dithiothreitol (150 mM), tunicamycin (2 μg/mL) or thapsigargin (100 nM), for 4 h. Xbp1 expression was evaluated by RT-PCR analysis. Polyacrylamide gel electrophoresis and subsequent ethidium bromide staining were performed to distinguish sXbp1 from uXbp1. The cropped image of RT-PCR analysis is shown because of space limitations; image of the full-length gel is Supplementary Fig. S7. (**B**–**D**) HB2 brown preadipocytes were treated with the indicated concentration of capsaicin for 4 h. Expression levels of sXbp1 (**B**), uXbp1 (**C**) or Chop (**D**) were examined by RT-qPCR analysis. The data are presented as the mean ± SE (n = 4). *P < 0.05 *vs*. control cells.

In response to ER stress, Xbp1 pre-mRNA is converted to spliced mRNA, leading to the production of the transcription factor Xbp1^21,22^. Thus, we evaluated the spliced form of Xbp1 (sXbp1) by RT-PCR in brown preadipocytes treated with capsaicin (Fig. 6A). Similar to known ER stress stimulators such as A23187, dithiothreitol, tunicamycin and thapsigargin^42^, supra-pharmacological capsaicin increased the expression of sXbp1.

We also examined sXbp1 expression by RT-qPCR. Previously, oligonucleotide primers for real-time qPCR were designed to discriminate human sXbp1 from unspliced Xbp1 (uXbp1)^43^; however, the PCR primers designed to the corresponding region of mouse Xbp1 recognized both sXbp1 and uXbp1 (Supplementary Fig. 4A). We therefore established a real-time qPCR system to separately evaluate sXbp1 and uXbp1 expression levels; the qPCR primers for sXbp1 exclusively recognized sXbp1, whereas those for uXbp1 exclusively recognized uXbp1 (Supplementary Fig. 4B,C). Furthermore, the amplification was not affected by the presence of unintentional Xbp1 (Supplementary Fig. 4D,E). We concluded that the primer sets specifically recognized the corresponding Xbp1 transcripts.

Expression of sXbp1 was significantly increased in brown preadipocytes treated with 100 μM capsaicin, although 10 μM capsaicin also slightly increased sXbp1 expression (Fig. 6B). The supra-pharmacological concentration of capsaicin also increased expression of Chop, a gene induced by ER stress^40,44^ (Fig. 5C). All these results demonstrated the induction of ER stress by the supra-pharmacological concentration of capsaicin in brown preadipocytes.

### Induction of ER stress stimulates brown adipogenesis

**Figure 7:**
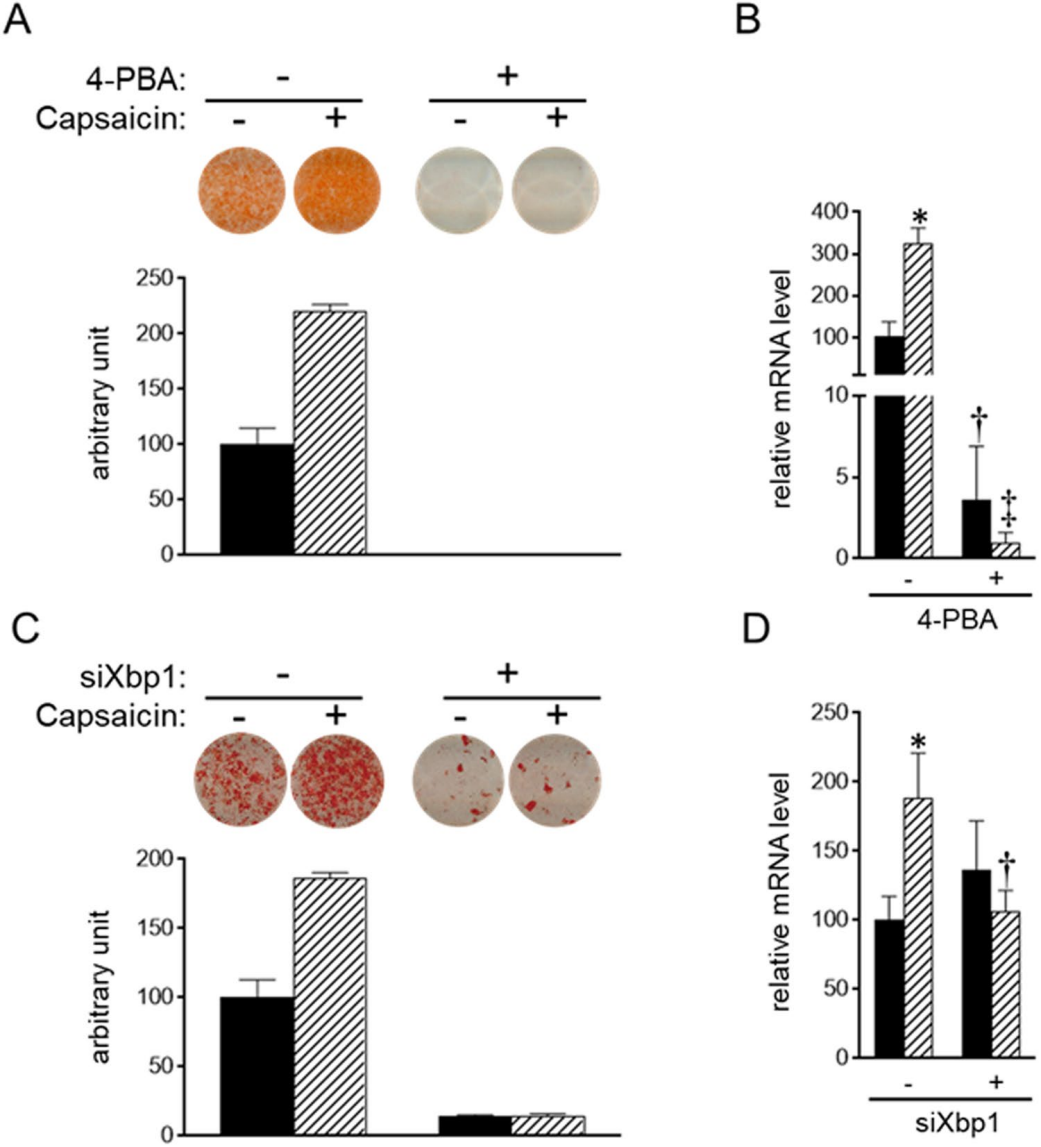
Requirement of ER stress for efficient brown adipogenesis. (**A** and **B**) HB2 brown preadipocytes were treated with or without 5 mM of 4-PBA or 100 μM of capsaicin or both for 8 days in the presence of insulin (20 nM). (**C** and **D**) HB2 brown preadipocytes were transfected with scrambled RNA or siRNA for Xbp1. Cells were treated with or without 100 μM of capsaicin in the presence of insulin (20 nM) for 8 days. (**A** and **C**) Oil Red O staining of cells on day 8 was performed, and the dye intensity was quantified (n = 2). (**B** and **D**) The expression level of Ucp1 was examined by RT-qPCR analysis. Black bar: vehicle; Hatched bar: capsaicin. The data are presented as the mean ± SE (n = 4). *P < 0.05 *vs*. cells treated with vehicle and corresponding reagent (vehicle or 4-PBA (**B**) or vehicle or siXbp1 (**D**)). ^†^*P* < 0.05 and ^‡^*P* < 0.01 *vs*. cells treated with vehicle and the corresponding reagent (vehicle or capsaicin).

To explore the role of supra-pharmacological capsaicin-induced ER stress, brown adipogenesis was evaluated in brown preadipocytes treated with 4-phenyl butyric acid (4-PBA), an inhibitor of ER stress^45^. Treatment with 4-PBA decreased lipid accumulation and Ucp1 expression on day 8, and capsaicin did not stimulate brown adipogenesis in 4-PBA-treated cells (Fig. 7A,B). We also examined the effect of down-regulation of Xbp1 expression; Xbp1 expression was decreased by transfection with siRNA for Xbp1, as expected (Supplementary Fig. 5). Consistent with the results in cells treated with 4-PBA, knockdown of Xbp1 gene decreased lipid accumulation on day 8 (Fig. 7C). However, it did not decrease Ucp1 expression (Fig. 7D). Capsaicin treatment did not increase lipid accumulation or Ucp1 expression in Xbp1-knockdown cells (Fig. 7C,D). These results suggested that ER stress is required for brown adipogenesis.

### Phosphorylation of cAMP-responsive element-binding protein (Creb) is stimulated by supra-pharmacological concentration of capsaicin

**Figure 8:**
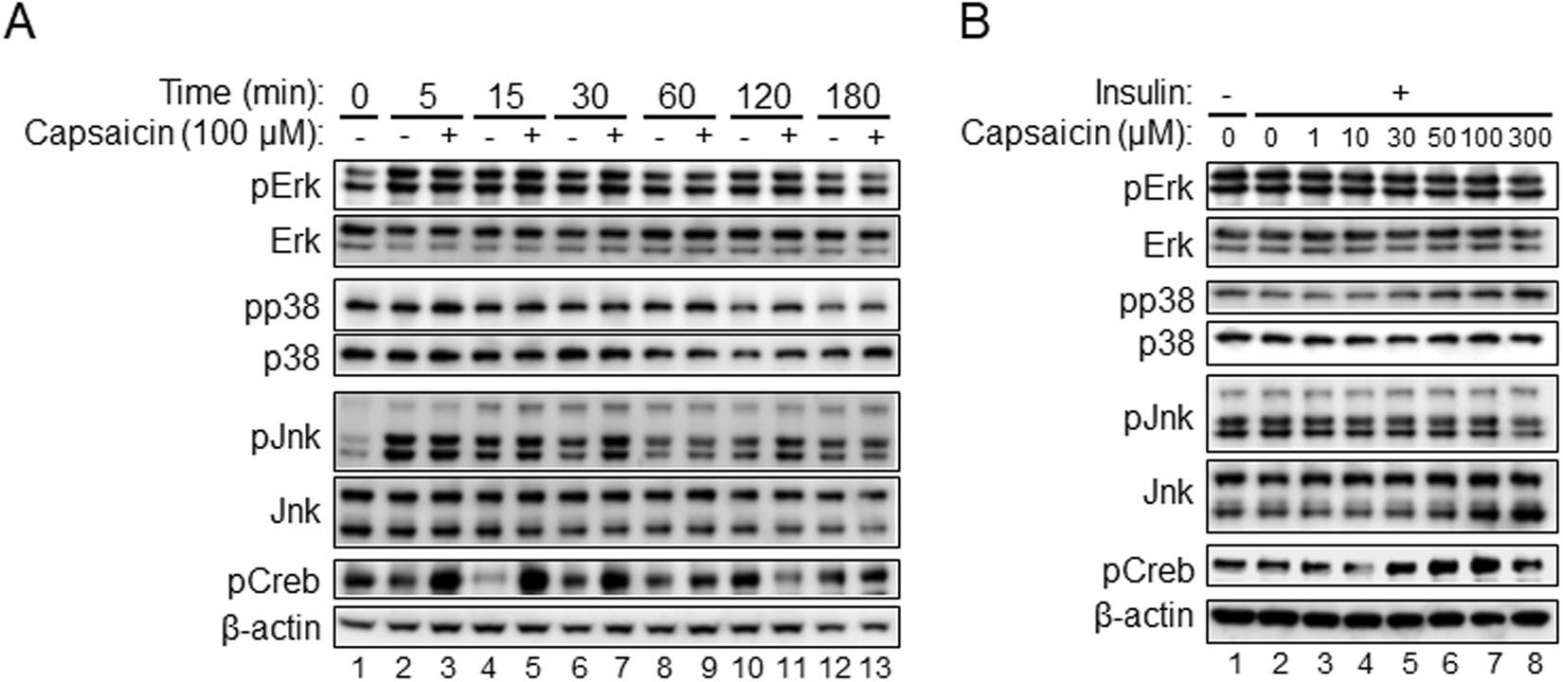
Stimulation of Creb phosphorylation by supra-pharmacological capsaicin. HB2 brown preadipocytes on day 0 were cultured with or without 100 μM capsaicin in the presence of insulin for the indicated time (**A**) or with the indicated concentration of capsaicin in the presence of insulin for 30 min (**B**). Expression level of phosphorylated (p) and total Erk, p38 and Jnk was examined by Western blot analysis. In addition, expression of pCreb as well as β-actin as the loading control was also evaluated. Representative results are shown. The cropped images of Western blot analysis are shown because of space limitations; images of the full-length blot are Supplementary Fig. S8 (**A**) and Fig. S9 (**B**).

We further evaluated possible signal cascade affected by the supra-pharmacological level of capsaicin. Previous studies showed mitogen-activated protein (MAP) kinases such as extracellular signal–regulated kinase (Erk), p38 and c-Jun N-terminal kinase (Jnk) are involved in various step of adipocyte differentiation^28,46–48^. Erk and Jnk were phosphorylated within 5 min after the differentiation stimulation (Fig. 8A, lanes 1 and 2), but 100 μM capsaicin did not affect Erk and Jnk phosphorylation. In contrast, neither the differentiation stimulation nor the supra-pharmacological level of capsaicin affected phosphorylation of p38. We also evaluated phosphorylation of Creb, because Creb is involved in activation of brown adipocytes^3,9,10^. Phosphorylation of Creb was rapidly and clearly detected in cells treated with 100 μM capsaicin (~5 min) (Fig. 8A, lanes 2 and 3), and treatment with capsaicin at the concentration of 30–100 μM effectively induced phosphorylation of Creb in a dose-dependent manner (Fig. 8B, lanes 2, 5–7).

## Discussion

Since the identification of functional brown adipocytes in adult humans^5–8^, many studies have explored the factors regulating brown adipogenesis and the function of brown adipocytes^9,10^. We previously revealed that capsaicin slightly stimulates the differentiation of brown preadipocytes in the late stages of brown adipogenesis, independent of sympathetic nerve activity^20^. Here, we demonstrated that 1) 100 μM capsaicin directly and stimulated the differentiation of brown preadipocytes in the early stages of brown adipogenesis, 2) capsaicin increased the concentration of cytosolic calcium through modulation of the intracellular calcium distribution, which presumably resulted in the induction of ER stress, 3) ER stress was required for brown adipogenesis, and 4) Creb was rapidly and clearly phosphorylated in response to the high level of capsaicin. So far, the stimulatory effect of capsaicin at the supra-pharmacological concentration on brown adipogenesis as well as the promotion of brown adipogenesis by ER stress is undetermined. The present results provided novel insights into the regulation of brown adipogenesis.

Previous studies revealed that 100 μM of capsaicin is extremely high, as this concentration induced growth inhibition and apoptosis in KB cancer cells, PC12 pheochromocytoma cells and AGC gastric carcinoma cells^49–51^. Considering that ER stress is well known to induce apoptosis^44,52^, the detrimental effects of 100 μM of capsaicin on cell growth is likely to be mediated by ER stress. In this study, 100 μM of capsaicin did not cause cell toxicity in brown preadipocytes but rather stimulated differentiation to brown adipocytes through the induction of ER stress. Previous studies revealed that cell differentiation was stimulated in myogenic cells, osteogenic cells and goblet cells of the intestine, when ER stress was mild^53–56^. We observed that treatment with tunicamycin or A23187, inducers of ER stress^42^ (Fig. 6A), during brown adipogenesis also increased lipid accumulation in brown adipocytes. However, these results were not reproducible, and the treatments frequently induced cell death (data not shown). It is possible that the intensity of ER stress has a critical role in brown adipogenesis. The present data demonstrated that 100 μM capsaicin reproducibly induced mild ER stress, leading to stimulation of brown adipogenesis.

ER stress has also been reported to modulate white adipogenesis^57–60^; however, this role of ER stress is controversial. Expression levels of genes related to ER stress such as sXbp1 and Chop were increased during the differentiation of white preadipocytes^57–60^, and down-regulation of Xbp1 expression or inhibition of ER stress by 4-PBA impeded adipogenesis^57–59^. These results suggested a potential role of ER stress as a stimulator for adipogenesis. In contrast, stimulation of ER stress by ER stress inducers, such as tunicamycin and hypoxia, decreased lipid accumulation and expression of genes related to adipocyte differentiation, suggesting an inhibitory role of ER stress in white adipogenesis^60^. These results suggest that ER stress has a dual role in white adipogenesis; the intensity of ER stress determines cell fate during white adipogenesis. Considering that the cell differentiation is regulated by the degree of ER stress in several biological systems, as shown above^54–56^, appropriate levels of ER stress, which are not too strong, may be required for proper development.

Capsaicin-induced calcium influx was previously shown to inhibit white adipogenesis^61–63^. However, the role of increased cytosolic calcium concentration in brown adipogenesis was unclear. Insulin primarily regulates brown adipogenesis and was shown to be essential for differentiation of brown preadipocytes^64,65^. Insulin is known to increase the cytosolic calcium concentration in some cells, such as white adipocytes and hepatocytes^66–68^. However, insulin did not increase the cytosolic calcium concentration in brown preadipocytes^69^ (data not shown). As stimulation of brown adipogenesis by insulin is thought to be mediated through a calcium-independent pathway, the enhancement of brown adipogenesis by supra-pharmacological capsaicin is thus mediated through an insulin-independent pathway.

Previous studies have revealed Trpv1-independent cellular responses to capsaicin; for example, capsaicin (~200 μM)-induced cell growth inhibition was not affected by Trpv1 antagonists in LNCaP and PC-3 prostate cancer cells^70^. In addition, capsaicin (100 μM) stimulated cell invasion, the release of insulin-like growth factor-1, and matrix metalloprotease-9 production in urothelial cancer cells that do not express Trpv1^71^. These results suggested that the supra-pharmacological effect of capsaicin is elicited through receptor(s) other than Trpv1. These Trpv1-independent capsaicin effects were detected when the dose of capsaicin was higher (≥100 μM). It is possible that capsaicin signals at least two types of receptors, i.e., a high-affinity Trpv1 receptor and an unidentified low-affinity receptor. Alternatively, high dose of capsaicin induced disruption of lipid bilayer membrane organization through its incorporation into phospholipids^72,73^. Thus, it is possible that the supra-pharmacological dose of capsaicin impairs function of membrane proteins, leading to increase in cytosolic calcium concentration.

We found that Creb phosphorylation was rapidly and clearly induced by the supra-pharmacological dose of capsaicin. Previous studies revealed that the increase in cytosolic calcium concentration led to phosphorylation of Creb^74,75^. Creb phosphorylation was also stimulated by induction of ER stress^76,77^. Future studies are needed to know how Creb is phosphorylated in response to the capsaicin as well as the role of phosphorylated Creb in brown adipogenesis.

Treatment with supra-pharmacological capsaicin for 12 hours after stimulated differentiation was sufficient to increase lipid accumulation in brown adipocytes, whereas longer treatment periods were required for up-regulation of Ucp1 expression. In addition, knockdown of the Xbp1 gene decreased lipid accumulation but not the expression level of Ucp1. Asada *et al*.^78^ showed that ER stress was induced in response to β3-adrenergic receptor activation in brown adipocytes. In addition, the induced sXbp1 was involved in the expression of Ucp1 in brown adipocytes, although the underlying mechanism was unclear^78^. Taken together, these findings indicate that the induction of ER stress may lead to acceleration of thermogenesis through stimulation of multiple processes in brown (pre)adipocytes. Future studies will be critical to understanding the mechanism underlying the effect of capsaicin on brown adipogenesis.

## Materials and Methods

### Materials

The reagents were purchased as follows: capsaicin, forskolin and thapsigargin were from Wako (Osaka, Japan); insulin was from Sigma (St. Louis, MO, USA); I-RTX was from Alomone Labs (Jerusalem, Israel); A23187 was from Cayman (Ann Arbor, MI, USA), Fluo-8-AM was from AAT Bioquest (Sunnyvale, CA, USA); 4-PBA was from Tokyo Chemical Industry (Tokyo, Japan); rabbit polyclonal antibody against Ucp1 (ab10983) and mouse monoclonal antibody against β-actin (AC-15) was from Abcam (Cambridge, MA, USA); rabbit polyclonal antibodies against phospho-Erk (Thr202/Tyr204) (#9101), Erk (#9102), phospho-p38 (Thr180/Tyr182) (#9211), p38 (#9212), phospho-Jnk (Thr183/Tyr185) (#9251), Jnk (#9252), and rabbit monoclonal antibody against phospho-Creb (Ser133) (87G3) were from Cell Signaling Technology (Danvers, MA, USA); goat polyclonal antibody against Parp-1 (A-20) was from Santa Cruz Biotechnology (Dallas, TX, USA); and Pluronic F-127 was from AnaSpec (Fremont, CA, USA).

### Animals and cell culture

Animal care and experiments were approved by the Animal Care Committee of Kyoto University (28–20), and all animal experiments were conducted in accordance with the approved guidelines. Mouse primary brown adipocytes were differentiated as previously described by Sharp *et al*.^79^; SV cells were isolated from interscapular brown adipose tissue of C57BL/6 mice using collagenase solution (1.5 mg/mL). SV cells plated in collagen-coated culture dishes were differentiated into brown adipocytes; the confluent cells grown in growth medium (Dulbecco’s modified Eagle medium (DMEM) containing 20% fetal bovine serum (FBS)) were cultured with differentiation medium (DMEM containing 10% FBS, 200 nM insulin, 0.25 mM isobutylmethylxanthine, 125 nM indomethacin, 1 mM dexamethasone, and 1 nM T3) for 2 days (day 0–2). Subsequently, cells were cultured with growth medium supplemented with 200 nM insulin and 1 nM T3. HB2 brown preadipocytes^80^, which were isolated from mouse interscapular brown adipose tissue and were kindly provided by Dr. M. Saito, Emeritus Professor of Hokkaido University, were cultured in DMEM with 10% FBS and antibiotics as previously described^20^. HB2 cells were differentiated into brown adipocytes by insulin (20 nM) two days after confluence (day 0) in the presence of capsaicin or vehicle (dimethyl sulfoxide). To evaluate gene induction in response to β-adrenergic receptor activation, brown adipocytes were treated with or without forskolin (10 μM) on day 8 for 4 h; β-adrenergic receptor activation stimulates protein kinase A pathway through increasing cAMP concentration^3^, and forskolin activates adenylate cyclase activity to increase cytosolic concentration of cAMP^30^.

### Dye imaging

Lipid accumulation was examined by Oil Red O staining on day 8; images were obtained by scanning stained wells (GT-9400UF, EPSON, Tokyo, Japan). The density of the dye was quantified from the image using ImageJ. Dye was also extracted with 2-propanol, and absorbance of the solution was measured at 510 nm for quantification^27^.

### Calcium imaging

The calcium imaging analysis was performed as previously described^20^; briefly, HB2 cells plated in 96-well plates were washed twice with phosphate-buffered saline with or without calcium, and loaded with 100 μl of the calcium imaging reagent, i.e., Fluo-8 AM and 0.04% Pluronic F-127, for 20 min at 37 °C. Cells were subsequently treated with capsaicin, A23187 or thapsigargin in the presence or absence of I-RTX and calcium. At least three independent experiments were performed, and representative results are shown.

### RNA isolation, RT-PCR and real-time RT-qPCR

Total RNA isolation was performed as previously described^20^. To evaluate the expression of sXbp1 and uXbp1, RT-PCR was performed as follows. The cDNA, reverse-transcribed from 5 ng of total RNA, was used as a template for RT-PCR. The oligonucleotide primers to detect both sXbp1 and uXbp1 were 5′-ACACGCTTGGGAATGGACAC-3′ and 5′-CCATGGGAAGATGTTCTGGG-3′. PCR was performed in a total volume of 10 μl containing 1 × KOD buffer with 1 mM MgCl_2_, 0.2 mM of each dNTP, 0.2 μM of each primer, and 0.2 U of a KOD DNA polymerase (Toyobo, Osaka, Japan). The PCR profile of RT-PCR is as follows: after denaturation for 2 min at 98 °C, 30 cycles consisting of 10 sec at 95 °C and 15 sec at 68 °C. The PCR products were separated in a 12% polyacrylamide gel in 1 × TBE and visualized with ethidium bromide.

Real-time RT-qPCR was performed as previously described^20^. The nucleotide sequence of qPCR primers for Ucp1,Pgc-1α, Pgc-1β, Cidea, Prdm16, Cox7α and TATA-binding protein (Tbp) were previously described^20,65,81^. The oligonucleotide primers for genes related to brown adipogenesis and function of brown adipocytes were as follows: 5′-ACTTCGAGACGTTTCAGGACTTA-3′ and 5′-GACGACCACTATGAGAAATGAGCTT-3′ for Elovl3, 5′-TGCCTTTACATCGTCTCCAA-3′ and 5′-GGCTCCAGGGTTCAGAAAGT-3′ for Cpt1β, 5′-AGTACCCTGTGGAGAAGCTGATG-3′ and 5′-TCAATGTGCTCACGAGCTATG-3′ for Mcad, 5′-TGCGAGAAGGCCTCTATTTCAAC-3′ and 5′-CATCCTTAGCTACTTGTGACATGGTA-3′ for CytoC, and 5′-TTTGGTGGCAGCGAGTCTAT-3′ and 5′-CCTGTATGGGGTTGCTCTTC-3′ for Cox4β. In addition, the oligonucleotide primers for sXbp1 were as follows: 5′-TCTGCTGAGTCCGCtaCAGGTG-3′ for the sense primer and 5′-CCATGGGAAGATGTTCTGGG-3′ as the antisense primer. The small characters in the sense primer indicate the mutations to the correct sequence, 5′-AG-3′, to prevent erroneous amplification of uXbp1. The nucleotide sequence of the sense primer for uXbp1 was 5′-CTCAGACTATGTGCACCTCTGC-3′, and the antisense primer for uXbp1 was the same as that for sXbp1. In addition, we designed oligonucleotide primers for sXbp1 based on the study by van Schadewijk *et al*.^43^; 5′-TGCTGAGTCCGCAGCAGGTG-3′ and 5′-ACTAGCAGACTCTGGGGAAG-3′, which were referred to as literature-based sXbp1 primers. The ΔΔCt method was used to normalize the levels of target transcripts to Tbp levels^82^.

### Plasmids

The cDNA of sXbp1 or uXbp1 gene was isolated from HB2 cells treated with 100 μM of capsaicin for 4 h and cloned into pcDNA3. The validity of the insert was verified by nucleotide sequencing.

### Western blot

Western blot analyses were performed as previously described^83^. The immunoreactive proteins were visualized using Chemi-Lumi One Ultra reagent (Nacalai Tesque, Kyoto, Japan) according to the manufacturer’s protocol.

### siRNA transfection

HB2 cells (1 × 10^4^ cells per well) were seeded onto 12-well plates. At confluence (day -2), cells were transfected with 6 μl of Lipofectamine RNAi Max (Thermo Fisher, Waltham, MA, USA) and 60 pmol of siRNA for Xbp1 or scrambled RNA (MISSION siRNA; Sigma) for 2 days. On day 0, siRNA was repeatedly transfected for 1 day. Capsaicin (100 μM) or vehicle was treated in the presence of insulin (20 nM) for day 0 to day 8. The nucleotide sequences of the double-stranded siRNA for Xbp1 are 5′-GAAUUCAUUGUCUCAGUGAdTdT-3′ and 5′-UCACUGAGACAAUGAAUUCdTdT-3′.

### Statistical analyses

Data are expressed as the mean ± standard error (SE). Data on gene expression were log-transformed to provide an approximation of a normal distribution before analyses. Differences from control values were examined using Dunnett’s test or unpaired *t*-test. When cells were treated with forskolin, 4-PBA or siXbp1, differences between cells under the same status of forskolin, 4-PBA or siXbp1, respectively, were examined by unpaired *t*-test. Differences of *P* < 0.05 were considered significant.

## Electronic supplementary material

Supplementary information
