# Supplementary material for: Supra-pharmacological concentration of capsaicin stimulates brown adipogenesis through induction of endoplasmic reticulum stress
Source: Sci Rep. 2018 Jan 16;8:845. doi: 10.1038/s41598-018-19223-2 (PMC5770457; doi:10.1038/s41598-018-19223-2)
Supplement: Supplementary file 1 — Supplementary information [file 41598_2018_19223_MOESM1_ESM.pdf]

Supplementary Information

Supra-pharmacological concentration of capsaicin stimulates brown adipogenesis through induction of endoplasmic reticulum stress

Ryosuke Kida, Taiki Noguchi, Masaru Murakami, Osamu Hashimoto,
Teruo Kawada, Tohru Matsui and Masayuki Funaba

Supplemental methods

Cell viability assay

HB2 cells (2×10^3 cells per well) were seeded onto 96-well plates. At 2 days after confluence (day 0), HB2 cells were treated with or without insulin (20 nM) and capsaicin at the indicated concentration for 2 days, or with or without actinomycin D (Act D, 1 μg/mL) in the presence of insulin (20 nM) for 1 day. Cell viability assays were performed using CellTiter-Glo® Luminescent Cell Viability Assay (Promega, Madison, WI USA), according to the manufacturer's protocol.

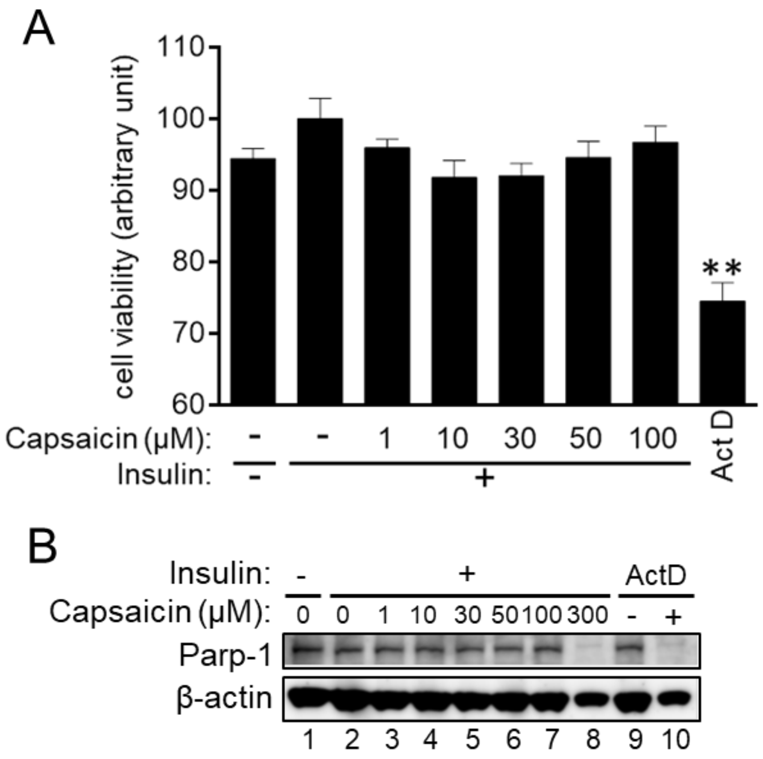

Figure S1. Cell viability and expression of full-length Parp-1 in response to supra-pharmacological capsaicin in HB2 cells

HB2 brown preadipocytes were cultured with or without the indicated concentration of capsaicin and insulin (20 nM) for 2 days. In addition, HB2 cells were treated with or without Act D (1 μg/mL) for 1 day (A) or 2 days (B). (A) Cell viability was evaluated, and ATP amount in control cells treated with insulin in the absence of capsaicin was set at 100. The data are presented as the mean ± SE (n = 4). **: $P < 0.01$ *vs.* control cells. (B) Parp-1 and β-actin were examined by Western blot analysis. The cropped images of Western blot analysis are shown because of space limitations; images of the full-length blot are Supplementary 9.

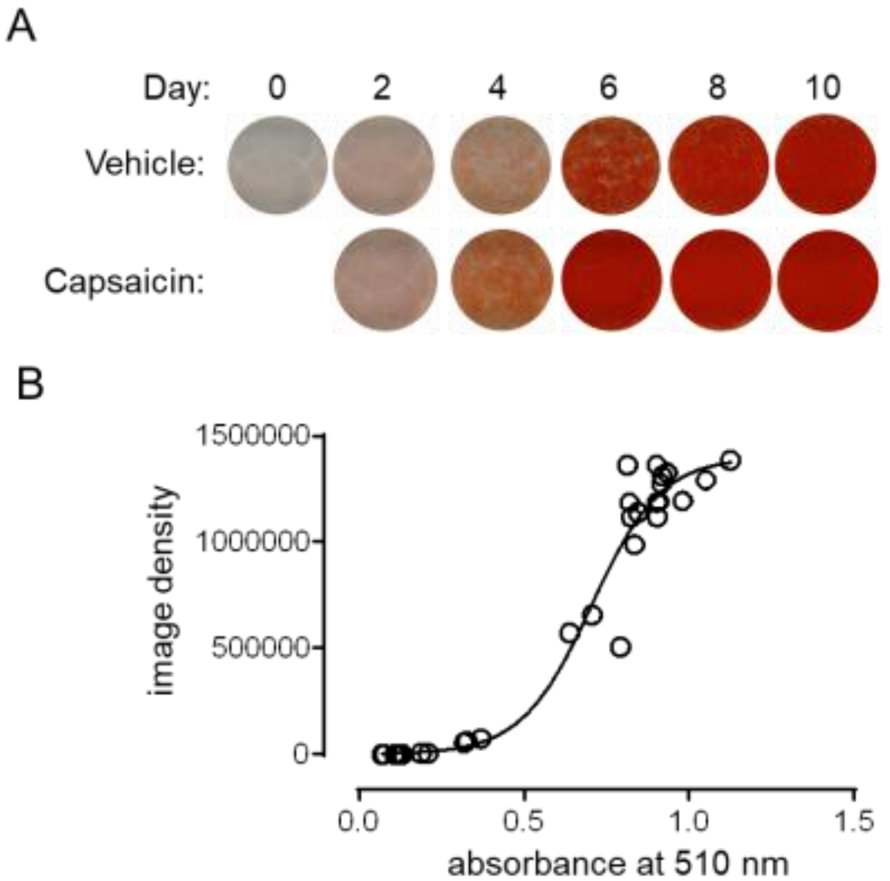

Figure S2. Time-course of changes in lipid accumulation in response to supra-pharmacological capsaicin in HB2 cells

HB2 brown preadipocytes were cultured with or without capsaicin (100 μM) in the presence of insulin (20 nM) for the indicated time. (A) Oil Red O staining was performed. (B) Dye intensity was evaluated by image analysis as well as colorimetric analysis after extraction with 2-propanol.

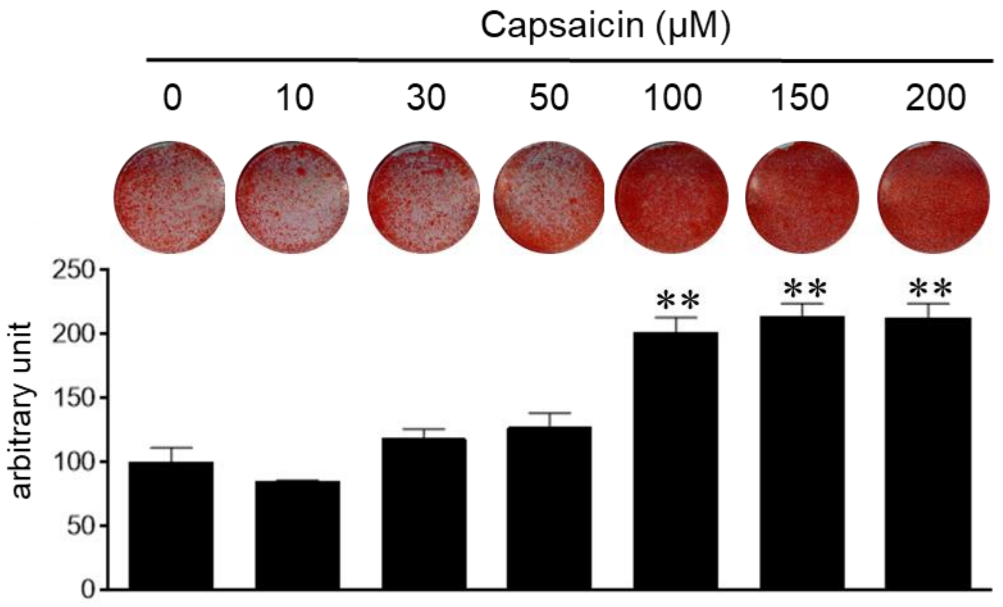

Figure S3. Effect of capsaicin concentration during brown adipogenesis on lipid accumulation

HB2 brown preadipocytes were cultured with the indicated concentration of capsaicin in the presence of insulin (20 nM) for 8 days. Oil Red O staining was performed, and dye intensity was evaluated by image analysis. A representative result is shown. The data are presented as the mean ± SE (n=3). **$P < 0.01$ *vs.* control cells.

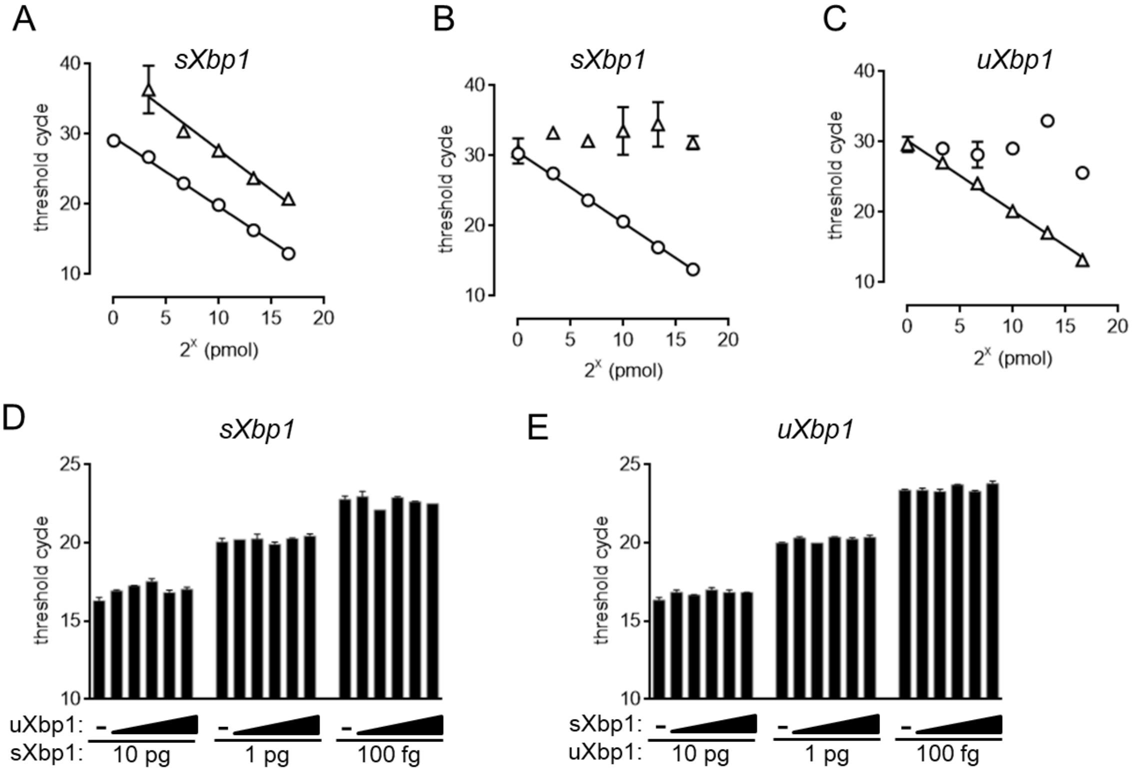

Figure S4. Determination of sXbp1 mRNA level by RT-qPCR analysis

(A-C) qPCR was performed using the indicated amount of plasmid encoding sXbp1 (circle) or uXbp1 (triangle) as a template. Threshold cycles were plotted against the amount of plasmid (n=2). (A) The sXbp1-directed primers based on the literature (van Schadewijk et al., 2012) were used. PCR efficiency for amplification of sXbp1 and uXbp1 was 98.9% and 114.1%, respectively. (B and C) PCR primers designed for sXbp1 (B) or uXbp1 (C) were used; the PCR efficiency for sXbp1 and uXbp1 was 100.7% and 99.9%, respectively. PCR primers for sXbp1 or uXbp1 did not amplify the opposite Xbp1 dose-dependently. (D and E) The effects of the presence of the unintentional Xbp1 were examined. The indicated amount of plasmid encoding sXbp1 (D) or uXbp1 (E) was amplified in the presence or absence (-) of various amounts (10 fg, 100 fg, 1 pg, 10 pg, 100 pg) of plasmid encoding uXbp1 (D) or sXbp1 (E) (n=2).

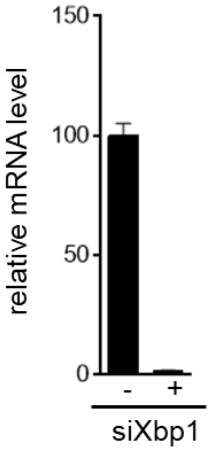

Figure S5. Xbp1 expression in HB2 cells transfected with siRNA for Xbp1

HB2 brown preadipocytes were transfected with scrambled RNA or siRNA for Xbp1. After 48 h, expression levels of total Xbp1 were examined by RT-qPCR analysis. The data are presented as the mean ± SE.

Fig. 1D

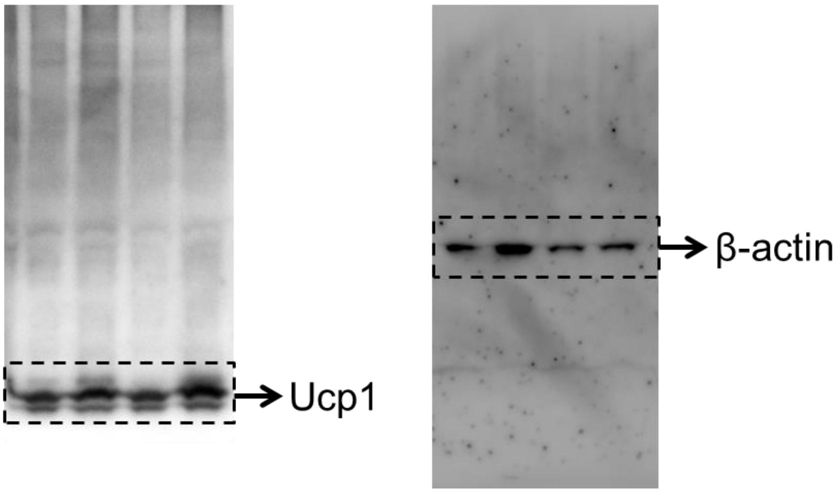

Figure S6. Images of full-length blot shown in Fig. 1D

Western blot analysis was visualized by chemical luminescence-based method. The raw results of Western blot analysis are shown. Dashed squares were cropped and shown in Fig. 1D.

Fig. 6A

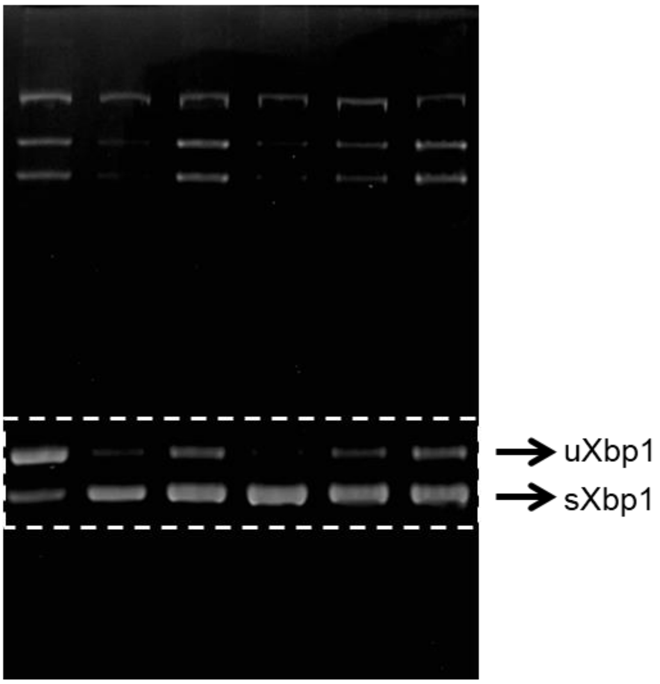

Figure S7. Images of full-length gel shown in Fig. 6A

RT-PCR was visualized by ethidium bromide staining. The raw results of RT-PCR analysis are shown. Dashed square was cropped and shown in Fig. 6A.

Fig. 8A

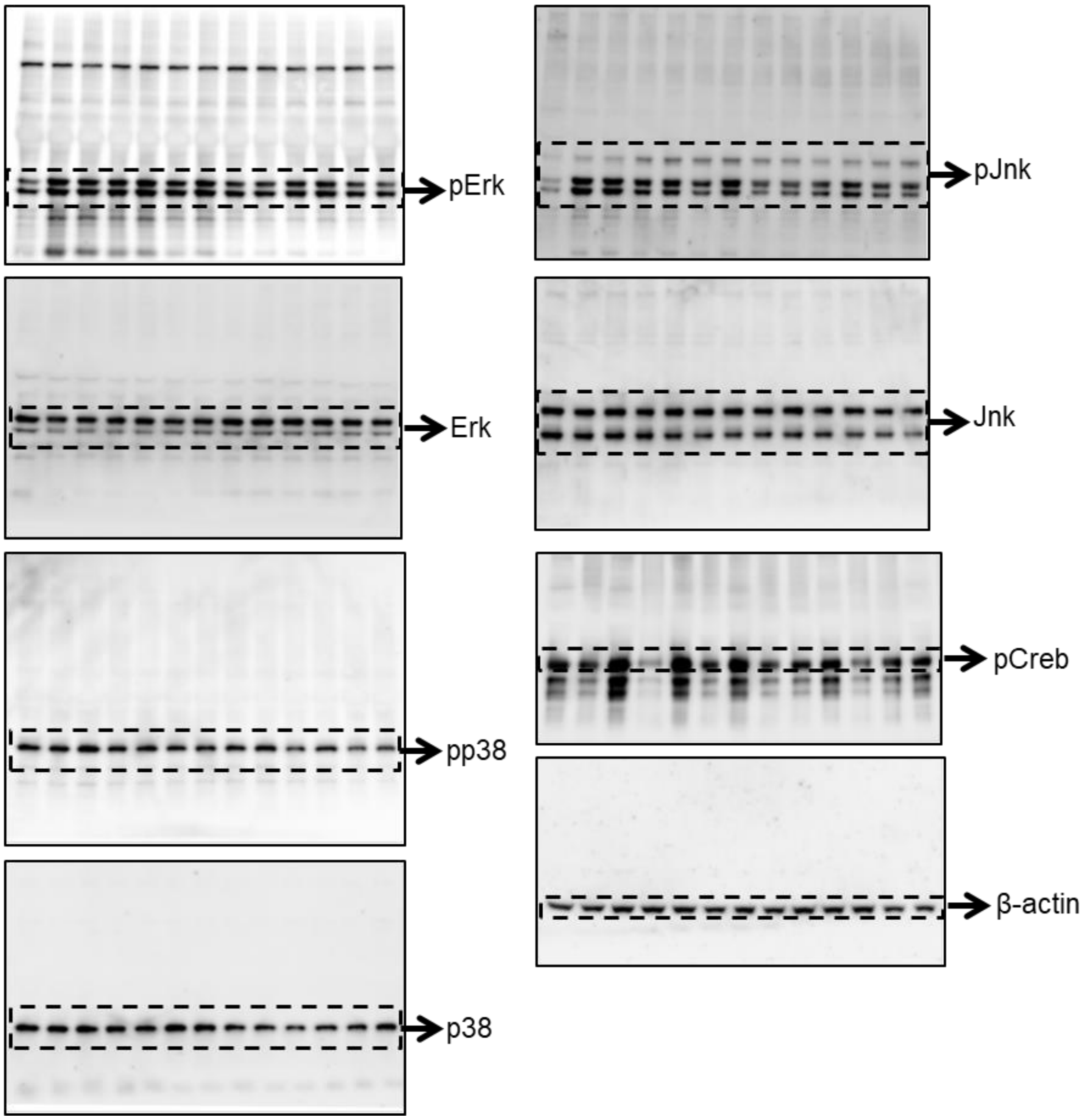

Figure S8. Images of full-length blot shown in Fig. 8A

Western blot analysis was visualized by chemical luminescence-based method. The raw results of Western blot analysis are shown. Dashed squares were cropped and shown in Fig. 8A.

Fig. 8B

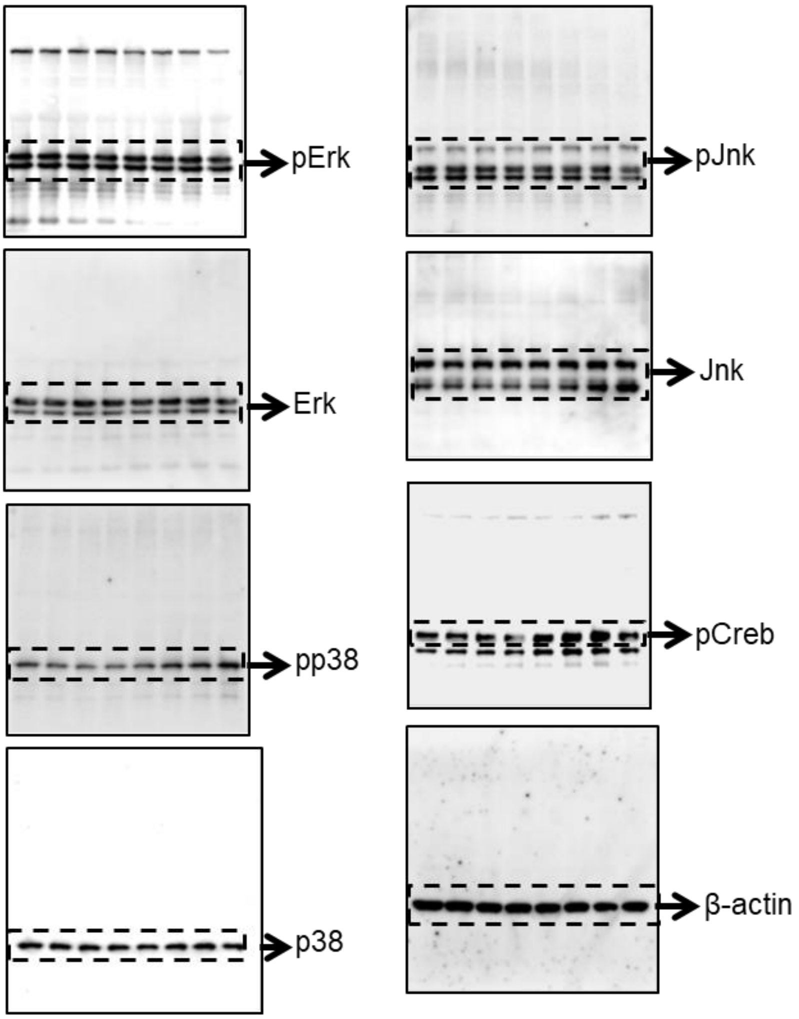

Figure S9. Images of full-length blot shown in Fig. 8B

Western blot analysis was visualized by chemical luminescence-based method. The raw results of Western blot analysis are shown. Dashed squares were cropped and shown in Fig. 8B.

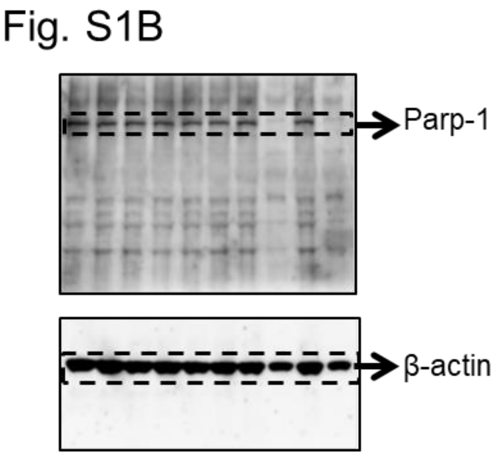

Figure S10. Images of full-length blot shown in Fig. S1B

Western blot analysis was visualized by chemical luminescence-based method. The raw results of Western blot analysis are shown. Dashed squares were cropped and shown in Fig. S1B.
